# Collaborative research on mental health in the post-COVID-19 era: an early career psychiatrists' perspective

**DOI:** 10.3389/fpsyt.2023.1230059

**Published:** 2023-08-24

**Authors:** Ramdas Ransing, Eugene Boon Yau Koh, Rodrigo Ramalho, Renato de Filippis, Mariana Pinto da Costa, Victor Pereira-Sanchez, Isa Multazam Noor, Mohammadreza Shalbafan

**Affiliations:** ^1^Department of Psychiatry, Clinical Neurosciences, and Addiction Medicine, All India Institute of Medical Sciences, Guwahati, Assam, India; ^2^Department of Psychiatry, Faculty of Medicine and Health Sciences, Universiti Putra Malaysia, Serdang, Malaysia; ^3^Department of Social and Community Health, School of Population Health, The University of Auckland, Auckland, New Zealand; ^4^Psychiatry Unit, Department of Health Sciences, University Magna Graecia of Catanzaro, Catanzaro, Italy; ^5^Institute of Psychiatry, Psychology and Neuroscience, King's College London, London, United Kingdom; ^6^Institute of Biomedical Sciences Abel Salazar, University of Porto, Porto, Portugal; ^7^Department of Child and Adolescent Psychiatry, New York University Grossman School of Medicine, New York, NY, United States; ^8^Department of Psychiatry, Faculty of Medicine, Yayasan Rumah Sakit Islam Indonesia (YARSI) University, Jakarta, Indonesia; ^9^Mental Health Research Center, Psychosocial Health Research Institute, Department of Psychiatry, School of Medicine, Iran University of Medical Sciences, Tehran, Iran

**Keywords:** mental health, mental disorders, psychiatry, collaborations, COVID-19 pandemic

The World Health Organization (WHO) has officially declared that COVID-19 is no longer a public health emergency, highlighting that this does not mean the disease is no longer a global threat (1). On the other hand, the mental health burden is expected to rise due to various factors, such as the global economic crisis, phenomena such as complicated grief and vaccine hesitancy, and the long-term consequences of COVID-19 itself (2, 3). The COVID-19 pandemic highlighted existing research and professional capacity gaps at a global level during a time when local preparedness and responses were critical (4). In turn, it also sped up the consolidation of existing collaborative networks of mental health professionals at a global scale and promoted the emergence of others (5). As per the Mental Health Preparedness and Action Framework (MHPAF), this is not the time to let our guard down (6). Instead, this is the time to reinforce and extend existing collaborations and develop further ones when needed in order to face the remaining threats of the COVID-19 pandemic and the rise of mental health needs.

Early-career psychiatrists (ECPs), connatural with technology and globalization, have been leading prominent efforts in terms of collaborative networks. Outstanding examples of this are the ECPs Section of the World Psychiatric Association (WPA), the World Network of Psychiatric Trainees (WNPT), and the Global Mental Health Think Tank (7, 8). During the COVID-19 pandemic, these collaborative networks served as powerful hubs for connection, peer support, brainstorming, and execution of academic collaborative ideas, synergies, and new, multidisciplinary teams (5). Furthermore, these international, web-based, multidisciplinary, and multicultural networks have been hubs for support, discussion, research, and action in perennial and emergent challenges of global psychiatry and mental health (5). They assisted ECPs in understanding the global state of mental health and mental healthcare in areas such as awareness, stigma, digitalization, inequities, racism, and discrimination (5). Their efforts led to numerous and impactful academic outputs related to COVID-19 and global mental health, such as protocols, guidelines, frameworks, and other scholarly publications and presentations (5).

The post-COVID-19 era presents an opportunity to continue to build beyond these existing networks. ECPs from every continent should be provided with an opportunity to explore and confront local and global challenges hand-in-hand with colleagues and friends worldwide. The return to in-person activities and the reduction of online work may reduce the frequency of remote virtual interactions and threaten the power of online-based networks. But there are clear opportunities to mix local and international collaboration and take advantage of in-person and at-a-distance opportunities via a “hybrid-new normal”. For example, while academic exchange programs have already started to pick up their previous pace (9, 10), we will probably see a more hybrid model after the pandemic, with in-person exchanges fulfilling a more targeted role within a larger scheme of virtual interactions (11). Still, this type of collaboration requires significant funding and resources to succeed. We urge governments, non-governmental organizations, professional associations, and philanthropic foundations to support and fund these collaborative research initiatives to sustain meaningful impact.

Overall, collaborative research on mental health in the post-COVID-19 era has the potential to improve our understanding of mental health conditions and develop effective interventions on a global scale. As members of global collaborative networks ourselves, we have identified five priority areas for research in the times to come (Table 1). The Mental Health Preparedness and Action Framework (MHPAF) was used to identify these research priorities (6).

**Table 1:** Research priorities for ECPs.

**No**	**Research priority**	**Justifications/examples**
1	Impact of COVID-19 on mental health in different communities and countries	Inequities regarding the current state of mental health and risk factors associated with mental disorders in different communities, countries, and regions.
2	Novel strategies to improve mental health and care	The COVID-19 pandemic saw new interventions and strategies aimed at improving mental health and care. Future research could assess their impact, including barriers and facilitators for developing and accessing these interventions. Recently developed and new interventions should be culturally sensitive and help address rather than reproduce existing inequities.
3	Digital mental health and care	The pandemic accelerated the adoption of digital technology in mental health care. Digital mental health and care may help improve access to quality, scalable, and cost-effective care. Future research should focus on the safety, feasibility, and acceptability of digitalization in mental health care.
4	Mental health-related stigma	There was increased mental health awareness during the pandemic, but further research could explore the impact of stigma.
5	Education and training	During the pandemic, micro-credentialing and micro-training were more accessible and known to improve clinical practice. There is a need to explore and assess the impact of this type of training, and whenever appropriate, expand educational opportunities, e.g., via online conferences and webinars, particularly for researchers from low and middle-income countries (LMICs).

Still, it is important to acknowledge that the potential rise of mental health issues in the near future is not the only threat we are facing at a global level. We also face the ongoing demand to address global challenges such as climate change, armed conflicts, and forced migrations. The COVID-19 pandemic has provided the opportunity for the world to unite with a shared goal, transcending local and national differences. We should continue moving forward in a similar spirit when facing these and any future challenge. In terms of international collective actions, not all global actors often prioritize global equity (12). However, the pandemic has proven the value of ensuring the active and fair participation of all actors—particularly the Global South, often the most affected by these threats—in the global discussion and collective action. A post-COVID-19 world would benefit immensely from building upon this learning.

## Author contributions

All authors listed have made a substantial, direct, and intellectual contribution to the work and approved it for publication.
